# Critical dynamics in associative memory networks

**DOI:** 10.3389/fncom.2013.00087

**Published:** 2013-07-24

**Authors:** Maximilian Uhlig, Anna Levina, Theo Geisel, J. Michael Herrmann

**Affiliations:** ^1^Bernstein Center for Computational NeuroscienceGöttingen, Germany; ^2^Max Planck Institute for Dynamics and Self-OrganizationGöttingen, Germany; ^3^Max Planck Institute for Mathematics in the SciencesLeipzig, Germany; ^4^Institute of Perception, Action and Behaviour, School of Informatics, University of EdinburghEdinburgh, UK

**Keywords:** associative memory, dynamical synapses, SOC, Hebbian learning, homeostatic learning

## Abstract

Critical behavior in neural networks is characterized by scale-free avalanche size distributions and can be explained by self-regulatory mechanisms. Theoretical and experimental evidence indicates that information storage capacity reaches its maximum in the critical regime. We study the effect of structural connectivity formed by Hebbian learning on the criticality of network dynamics. The network only endowed with Hebbian learning does not allow for simultaneous information storage and criticality. However, the critical regime can be stabilized by short-term synaptic dynamics in the form of synaptic depression and facilitation or, alternatively, by homeostatic adaptation of the synaptic weights. We show that a heterogeneous distribution of maximal synaptic strengths does not preclude criticality if the Hebbian learning is alternated with periods of critical dynamics recovery. We discuss the relevance of these findings for the flexibility of memory in aging and with respect to the recent theory of synaptic plasticity.

## 1. Introduction

Critical dynamics in neural networks is an experimentally and conceptually established phenomenon which has been shown to be important for information processing in the brain. Critical neural networks have optimal computational capabilities, information transmission and capacity (Beggs and Plenz, 2003; Haldeman and Beggs, 2005; Chialvo, 2006; Shew and Plenz, 2012). At the same time the theoretical understanding of neural avalanches has been developed starting from sandpile-like systems (Herz and Hopfield, 1995) and homogeneous networks (Eurich et al., 2002), but later also including particular structural connectivity (Lin and Chen, 2005; Teramae and Fukai, 2007; Larremore et al., 2011). The network structure in the latter cases was, however, chosen as to support or even to enable criticality, which points obviously to one of the mechanisms criticality is brought about in natural systems. There are, nevertheless, other influences that shape the connectivity structure and weighting. Most prominently, this includes Hebbian learning, but also homeostatic effects or pathological changes. Here we study how such structural changes influence criticality in neural networks.

While homeostatic plasticity may well have a regulatory effect that supports criticality, this cannot be said about Hebbian learning which essentially imprints structure from internally or externally caused activation patterns in the synaptic weighting of the network increasing thus the probability of previous patterns to reoccur. Unless the patterns are carefully chosen to produce critical behavior, these effects have a tendency to counteract critical behavior, e.g., by introducing a particular scale that corrupts the power-law distributions characteristic for critical behavior.

Little is known, in particular, about the influence of criticality on associative memory neural networks. We have chosen this paradigm of long-term memory as a basis for the present model because it is very well understood and because it matches the complexity of models that are typically considered in the study of criticality. Associative memory networks are able to recall stored patterns when a stimulus is presented, that is similar to one of the stored patterns, thus providing a means to implement memory into a neuronal population. If all goes well, the network state follows an attractor dynamics toward the correct memory item when being initialized by a corrupted or incomplete variant of the item as an associative key. Items are stored as activation patterns that are implanted in the network by Hebbian learning. This leads to an effective energy landscape, where the patterns are local minima and as such attractors of the system dynamics (Hopfield, 1982; Herz and Hopfield, 1995). We have studied earlier the effect of dynamical synapses (Markram and Tsodyks, 1996) in associative memory networks (Bibitchkov et al., 2002), now we are interested in the criticalizing role of dynamical synapses.

Other work has shown (Levina et al., 2006, 2007b; Levina, 2008; Levina et al., 2009) that dynamical synapses play an important role in the self-organization of critical neural dynamics. Given the importance of the critical regime for information processing in the brain and the substantial experimental evidence that is available to date, there is a need to consider the compatibility of these two effects and to identify a way to obtain criticality and memory storage simultaneously.

We will discuss here an algorithm to achieve compromise between a critical dynamics that can be seen as exploring the spaces of neural activation patterns, and the attractor dynamics that we assume to underlay the retrieval of content from memory. The present paper continues upon earlier work (Schrobsdorff et al., 2009; Dasgupta and Herrmann, 2011), where the preliminary simulation results were discussed. In our study for the first time conclusive numerical representations are presented, several learning mechanisms are compared and the capacity limit is considered.

## 2. Materials and methods

### 2.1. Neuronal activity dynamics in the critical regime

we consider a network of *N* integrate and fire neurons. The membrane potential *h*_*i*_ ∈ [0, θ] of a neuron *i* ∈ {1, …, *N*} is subject to the dynamics

(1) $${\dot{h}}_{i}=I_{\text{ext}}\delta \left(t-t_{\text{e}}^{i}\right)+\frac{1}{N}\sum_{j=1}^{N}J_{ij}\delta \left(t-t_{\text{sp}}^{j}\right).$$

The first term on the right hand side of Equation 1 represents an external excitatory input of size *I*_ext_ affecting neuron *i* at time *t*^*i*^_*e*_. The second term describes a recurrent excitatory input, where *t*^*j*^_sp_ denotes the arrival time of a presynaptic action potential originating from neuron *j* and *J*_*ij*_ is the strength (or weight) of the synapse connecting *j* to *i*. Action potentials are generated and delivered to all postsynaptic neurons when neuron *i* reaches the membrane potential threshold θ. After this depolarization, the potential is reset according to

(2) $$h_{i}\left(t_{\text{sp}}^{+}\right)=h_{i}\left(t_{\text{sp}}\right)-\theta .$$

The activity dynamics in this model network depends on the connectivity and the weight matrix *J* = {*J*_*ij*_}. For a fully connected network with equal weights the activity forms a series of avalanches that are separated by longer periods of quiescence (Eurich et al., 2002). An avalanche is triggered when external input *I*_ext_ causes a neuron to fire and consists of a number (the avalanche *size L*) of successive depolarizations. Because some of these activations may occur simultaneously, avalanches are also characterized by their *duration* (*D*), i.e., the time from the start of the avalanche to the firing time of the last neuron that was activated in this way.

For neural networks of this type a critical synaptic weight *J*_cr_ exists that leads to a scale-free avalanche size distribution *P*(*L*) (Eurich et al., 2002). For more complex networks the critical value is usually not explicitly obtainable, except for random (Levina et al., 2007b) or regularly coupled networks (Herz and Hopfield, 1995). This problem can be circumvented by applying an adaptive algorithm that adjusts the weights toward their critical values which do not need to be identical across neurons. Such an adaptation toward criticality can be obtained in form of a homeostatic learning rule (Levina et al., 2007a) which locally regulates the flow of activity from a neuron to its postsynaptic partners. Within the branching process approximation (Otter, 1949; Beggs and Plenz, 2003; Levina et al., 2007a; Levina, 2008) it can be shown that this homeostatic rule causes the network to become critical such that the activity dynamics in the network together with the homeostatic regulation forms a self-organized critical system.

### 2.2. Homeostatic regulation

Self-organized criticality can be achieved by applying a homeostatic learning rule at the beginning of each avalanche (Levina et al., 2007a) according to

(3) $$J_{ij}=J_{ij}^{0}+\varepsilon_{\text{hom}}\left[1-\ell -N^{-\frac{1}{2}}\right].$$

Here, *J*^0^_*ij*_ denotes the synaptic weights at the time of avalanche initiation, ℓ is the number of active neurons in the second time step of the avalanche, ε_hom_ is a learning rate and $N^{-\frac{1}{2}}$ a finite size correction. According to Equation 3 in the limit *N* → ∞, the synaptic weights will decrease if ℓ > 1, and increase if ℓ < 1. For an infinitely large *N* a stable configuration is obviously obtained as soon as the triggering neuron causes exactly one other neuron to fire. This corresponds to a mathematical model of a critical branching process, that is known to result in a power-law distribution of the avalanche size. A finite size correction is needed because avalanches cannot spread to infinity but are rather limited to a system of *N* neurons. Such a learning rule resembles the homeostatic regulation observed in cortical neurons (Abbott and LeMasson, 1993; Turrigiano et al., 1998), with the important difference that the neuron does not keep stable its own firing rate, but that of the postsynaptic population.

### 2.3. Dynamical synapses

The empirical observation of criticality in networks of real neurons has initiated a number of alternative explanations by regulatory processes interacting with the neuronal activity dynamics. One mechanism relies on the short-term dynamics of synaptic efficacies (Tsodyks and Markram, 1996, 1997). Given that synaptic resources are limited, high activity of presynaptic neurons will lead to depletion of these resources and thus to a reduced synaptic efficacy. In periods of silence or low activity, the synaptic efficacy will then recover toward its maximum value *T*^max^_*ij*_. We have modeled this in the following way (Levina et al., 2007b),

(4) $$\dot{T_{ij}}=\frac{1}{\tau_{J}}\left(T_{ij}^{\text{max}}-T_{ij}\right)-uT_{ij}\delta \left(t-t_{\text{sp}}^{j}\right),$$

where τ_*J*_ sets the time scale of exponential recovery, *t*^*j*^_sp_ is the presynaptic spike time and *u* sets the relative amount of resources used upon spike transmission (Markram and Tsodyks, 1996). Note that the *T*_*ij*_ are not the synaptic weights *J*_*ij*_ used in the sense of the previous section, but are related to Equation 3 by *J*_*ij*_ = *uT*_*ij*_. The *T*^max^_*ij*_ can be considered to be equal for all the synapses in the network and constant in time, but we will later relax this condition by introducing learning effects on a short time scale. Intuitively, the stabilizing effect of dynamical synapses in this model can be understood in the following way: large avalanches lead to depletion of synaptic resources and thus to series of smaller events, whereas small avalanches lead to an increase of the amount of resources in the synapses resulting in larger avalanches again. Such activity dependent regulation allows for a power-law distribution of avalanche sizes. A mathematical explanation for the success of this model is provided by the fact that 〈*uT*_*ij*_〉 → *J*_cr_ for a wide range of *T*^max^, i.e., the time-averaged synaptic input approaches the critical value *J*_cr_ of the network with static synaptic weights that was defined in section 2.1 (Eurich et al., 2002; Levina et al., 2007b).

The arrival times *t*^*i*^_*e*_ of external inputs of strength *I*_ext_ in Equation 1 are determined by a random process that selects neurons at a rate τ and increases their membrane potential. The characteristic time scale of synaptic recovery τ_*J*_ is related to the time scale of external input τ via τ_*J*_ = τν*N* and 1 < ν ≪ *N*. Therefore, the synaptic dynamics of this model is composed of two regimes. In the slow regime, neurons get loaded by external input *I*_ext_ and synaptic resources slowly recover toward their maximum value *T*^max^_*ij*_. The activation of a single neuron then marks the transition to the fast “avalanche regime”, where the redistribution of neuronal membrane potentials and depression of resources *T*_*ij*_ is so fast that we can safely assume external input and synaptic recovery processes to be absent.

Irrespective of the particular recipe used to achieve self-organized criticality in our simulations, we always record *A*_ava_ avalanches and calculate the mean squared deviation Δγ between the resulting avalanche size distribution *P*(*L*) and the best-matching power law over the range 1 ≤ *L* ≤ *N*/2. Unless Δγ is not less than a specified threshold Δγ^max^, we keep recording *A*_ava_ avalanches until the resulting size distribution has converged toward a power law and this sets the end of the critical regime. The resulting synaptic weight configuration {*J*_*ij*_} does then represent a neural network operating at the critical point. For small network sizes Δγ was shown to be as informative about criticality in the network as a Kolmogorov-Smirnov statistic with Monte-Carlo generated *p*-value (Levina, 2008).

### 2.4. Associative memory model

So far we have described the dynamics of the neural network in the critical regime. We now equip the network with the ability to store a set of patterns and to operate as an associative memory of these patterns. The patterns are represented by differences among the synaptic efficacies, and the retrieval of the pattern is understood as an attractor dynamics from a cue toward the pattern. The cue is a stimulus that causes a neuronal activity pattern near one of the memorized patterns and once the stimulus has initiated the attractor dynamics, it is expected that the current activity approaches the memorized pattern even more closely.

Let {ξ^η^}, η = 1, …, *M*, be a set of binary patterns consisting of pixels ξ^η^_*i*_ ∈ {0, 1}. The pattern ξ^η^ is retrieved if the firing rate of the neurons with ξ^η^_*i*_ = 1 is above and of the neurons with ξ^η^_*i*_ = 0 is below a certain threshold.

We assume a sparse representation, i.e., only a fraction *p* of the neurons in a pattern is assumed to be active such that for all η

(5) $$\frac{1}{N}\sum_{i=1}^{N}\xi_{i}^{\eta }=p.$$

In order to imprint these *M* binary patterns on the network connectivity, a matrix *W* = {*W*_*ij*_} in a correlational form is defined as

(6) $$W_{ij}=\frac{1}{p(1-p)\;N\;C}\sum_{\eta =1}^{M}\xi_{i}^{\eta }\xi_{j}^{\eta }\left(1-\delta_{ij}\right),$$

where *C* is an additional scaling factor which we choose such that ∑_*ij*_*W*_*ij*_ = *N*. The structure of this matrix is fixed in time and depends on the specific set of patterns. If we took the synaptic weight matrix in the same way, i.e., {*J*_*ij*_} = {*W*_*ij*_}, the network would exhibit optimal retrieval quality for the stored patterns η (Tsodyks and Feigel'man, 1988; Tsodyks, 1989). However, this weight configuration cannot be expected to generate critical avalanche size distributions. In order to combine criticality and memory, we therefore start with synaptic weights obtained by homeostatic learning (or dynamical synapses) and then carefully push the {*J*_*ij*_} toward the configuration {*W*_*ij*_} using the learning rule

(7) $$J_{ij}(t+1)=J_{ij}(t)+\varepsilon_{\text{hebb}}\left[W_{ij}-J_{ij}(t)\right].$$

Here, *J*_*ij*_(*t*) and *J*_*ij*_(*t* + 1) are respectively the old and the new synaptic strengths and ε_hebb_ ≪ 1 is a learning rate. Note that we do not apply Equation 7 synchronously for all the synapses but rather in a stochastic manner with update probability *p* = 1/*N* for each synapse (*i*, *j*). This is done until the synaptic weight configuration {*J*_*ij*_} allows for associative recall of the stored patterns as specified below. We will refer to the episode during which Equation 7 is applied as *Hebbian learning*.

A modified learning rule is implemented in the case of dynamical synapses, which is given by

(8) $$T_{ij}^{\text{max}}(t+1)=T_{ij}^{\text{max}}(t)+\varepsilon_{\text{hebb}}\left[u^{-1}W_{ij}-T_{ij}^{\text{max}}(t)\right].$$

Unlike before, Hebbian learning is not applied to the instantaneous values of synaptic efficacies {*T*_*ij*_} but rather to the maximal efficacies $\left\{T_{ij}^{\text{max}}\right\}$. Learning of instantaneous efficacies is not reasonable here as the effect of learning would be erased during the critical episode because the {*T*_*ij*_} tend to closely recover to their maximum values and these do not contain information about the stored patterns. If, however, the $\left\{T_{ij}^{\text{max}}\right\}$ are structured in a way similar to the optimal memory configuration {*W*_*ij*_}, the instantaneous efficacies {*T*_*ij*_} will be affected in favor of the memory configuration too because they recover during episodes of low network activity toward the $\left\{T_{ij}^{\text{max}}\right\}$.

Clearly, we need a criterion that sets the end of the Hebbian learning episode. This criterion can only be based on the retrieval quality of the current network state, which is discussed in the following section.

### 2.5. Retrieval quality

In order to assess the retrieval (or memory) quality in the network with the configuration of synaptic strengths {*J*_*ij*_(*t*)}, we construct perturbed versions κ^η^ = *Q*ξ^η^ of the stored patterns. The operator *Q* selects an active and an inactive neuron at random and swaps their states, thereby keeping the total number of active neurons unchanged. Ideally, the network will be able to reconstruct the original ξ^η^ from the κ^η^ using the information that is implicitly stored in the connections. Practically, we can only require that the network produces a state that has less errors than κ^η^, i.e., that is closer to the stored pattern than the perturbed version.

In discussing these questions, we will use a simplified model which was chosen mainly in order to be able to relate to results in Levina et al. (2007b, 2009) as well as in Bibitchkov et al. (2002). In addition to the use of binary patterns we will assume a noise-free dynamics during retrieval and a fixed threshold. The threshold is optimized for achieving maximal overlap in the next state which, however, does not imply an optimal retrieval in the convergent phase (Bibitchkov et al., 2002). More specifically, upon presenting a perturbed pattern κ^η^, the network activity will switch to a new configuration *S*^η^ given by

(9) $$S_{i}^{\eta }\left(\kappa^{\eta },\;\Theta \right)=\text{sign}\left(\sum_{j}J_{ij}\kappa_{j}^{\eta }-\Theta \right),$$

with Θ being some threshold. In what follows, we will refer to *S*^η^ as the *retrieved pattern*. To quantify the overlap between two binary patterns ξ and κ we use the correlational measure

(10) $$o(\xi ,\;\kappa )=\frac{1}{N}\frac{\sum_{i=1}^{N}\left[\left(\xi_{i}-m_{\xi }\right)\left(\kappa_{i}-m_{\kappa }\right)\right]}{\sigma_{\xi }\sigma_{\kappa }},$$

where *m*_ξ_ and *m*_κ_ denote the mean activities and σ_ξ_ and σ_κ_ are the standard deviations, respectively. Perfect overlap is obtained for identical patterns where we have *o*(ξ, κ) = 1, while we obtain *o*(ξ, κ) = 0 for uncorrelated patterns. Thus, the observation that

(11) $${\langle o\left(S^{\eta },\;\xi^{\eta }\right)\rangle }_{\xi }-{\langle o\left(\kappa^{\eta },\;\xi^{\eta }\right)\rangle }_{\xi }>\Delta ,$$

where Δ is a positive value that sets the minimum required improvement in (average) overlap 〈o (*S*^η^, ξ^η^)〉_ξ_ compared to the perturbations, provides evidence that the weight configuration {*J*_*ij*_} indeed contains information about the stored pattern ξ^η^. If the network realized, in contrast, an identical transformation it would not achieve an improvement of the overlap, but it could “remember” a pattern for a short time in a kind of short-term memory. Typically, we will not only consider a single random perturbation κ^η^ per pattern but many, so that Equation 11 becomes

(12) $${\langle {\langle o\left(S^{\eta },\;\xi^{\eta }\right)\rangle }_{\kappa }\rangle }_{\xi }-{\langle {\langle o\left(\kappa^{\eta },\;\xi^{\eta }\right)\rangle }_{\kappa }\rangle }_{\xi }>\Delta .$$

In a spiking network also temporal averages need to be included in order to obtain a consistent measurement of the retrieval quality. According to the simplifying assumptions above, we will consider only small perturbations which consist in the case of a finite network in single bit swaps. This is done for two reasons. First, such perturbations are used in order to concentrate on the threshold-independent effects of the retrieval dynamics. A perturbed pattern cannot be corrected by the choice of a standard threshold value. Second, near the critical capacity, it is sufficient to study the ability of the network to correct a single error. This is due to the reduction of the size of the basins of attraction of the pattern-related attractor states. As soon as the attractor size has reached zero even an almost correct pattern will typically deteriorate with the dynamics (Equation 9). A persistence of a fixed point state beyond the capacity limit, but without a basin of attraction, is easily achieved, e.g., by avoiding any update of the neurons state, but is not interesting in the present context.

Two comments concerning the threshold Θ in Equation 9 seem to be in order here. First, we choose this threshold such that the average overlap ${\langle {\langle o\left(S^{\eta },\;\xi^{\eta }\right)\rangle }_{\kappa }\rangle }_{\xi }$ is maximal. Second, Θ may differ from the threshold θ that we use in the critical regime. We assume that both thresholds are the result of a specific action of inhibitory neurons, which we, however, do not model here explicitly.

### 2.6. Optimization toward converged states

In the previous sections we have outlined how the synaptic weights evolve during the critical and Hebbian learning episodes, respectively. The critical episode ends as soon as the sampled avalanche size distribution is close to a power law (see section 2.3), whereas the following phase of Hebbian learning is stopped as soon as the network exhibits good retrieval quality of the stored patterns, when perturbations of the latter are presented. We measure the retrieval quality after each step of Hebbian learning and stop if the improvement in average overlap is at least Δ_hebb_. For the sake of reduced numerical complexity, we only consider one single perturbation for each stored pattern, i.e., we use Equation 11 instead of Equation 12. After Hebbian learning is over, the network is driven toward the critical regime again, employing either homeostatic regulation of synaptic weights or dynamical synapses, respectively. This alternation between the two episodes may be interpreted as an optimization scheme and the delicate question is if convergence toward a state is obtained in the long run, in which the network is critical and retains an associative memory of the stored patterns at the same time. In what follows, we will refer to these states as *converged states*.

Whether the network is in a converged state is always checked after the critical episode is finished and before the next round of Hebbian learning is started. At this point we already know that the system is operating at the critical point but we still need to make sure, that the critical episode has not erased memory of the stored patterns. We therefore rigorously test the retrieval quality of the network using Equation 12 with a minimum required improvement of Δ_conv_ and take the average over *n*_*p*_ (*n*_*p*_ ≫ 1) perturbations per pattern. Note that in the case of dynamical synapses, we assess the retrieval quality of *J*_*ij*_ = *uT*^max^_*ij*_ in the Hebbian learning phase, whereas we take *J*_*ij*_ = *uT*_*ij*_ to check for convergence.

**Figure 1:**
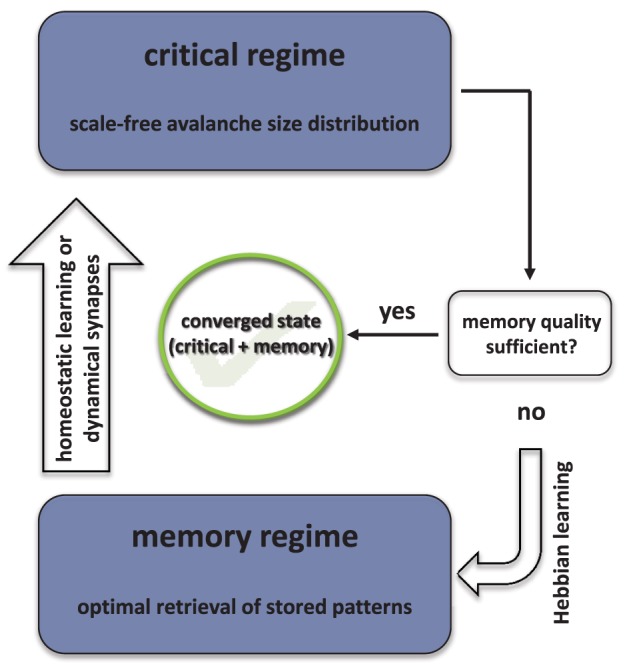
**Schematic representation of the dual optimization algorithm**.

A sketch of the optimization strategy is shown in Figure 1 and the most important steps are summarized in Algorithm 1 for the case of homeostatic regulation.

**Algorithm 1:** **General steps of the optimization strategy for the case of homeostatic regulation (see text for details)**.

Homeostatic learning of synaptic weights (Equation 3) based on *T*_L_ avalanches. Recording of *A*_ava_ avalanches and their sizes *L*. Calculate mean squared deviation Δγ of size distribution *P*(*L*) from best-fit power law; if Δγ < Δγ^max^ continue, otherwise restart at step 1. Check for convergence using Equation 12 with Δ = Δ_conv_ and averaging over *n*_*p*_ (*n*_*p*_ ≫ 1) perturbations per pattern; if retrieved states show large enough overlap with stored patterns, network has converged; if not, continue with step 5. Hebbian learning of synaptic weights using Equation 7; after each learning step check retrieval quality according to Equation 11 with Δ = Δ_hebb_; if retrieval quality good enough start at step 1.

## 3. Results

### 3.1. Specifications of the model used in the experiments

In this study we always simulate networks of *N* = 300 neurons. Other parameters are summarized in Table 1.

**Table 1:** **Parameters used in the numerical simulations**.

**General parameters**										
Parameter	*N*	θ	*p*	*A*_0_	*A*_ava_	ε_hebb_	Δγ^max^	Δ_hebb_	Δ_conv_	*n*_*p*_
Value	300	1.0	0.1	10^4^	10^6^	0.01	0.005	0.035	0.03	1000
**Homeostatic plasticity**							**Dynamical synapses**			
Parameter	*T*_L_	ε_hom_	*I*_e_				Parameter	ν	*u*	*I*_e_
Value	10^3^	0.001	0.0067				Value	10	0.2	0.025

At the beginning of each critical phase we sample *A*_0_ avalanches without taking them into account in the avalanche size distribution *P*(*L*). This is done to ensure that the size distribution is not affected by transient dynamics. The sampling of the distribution is an important contribution to the total simulation time and was the main limitation of the size of the network. Because in larger networks also larger avalanches need to be considered, the sampling time for given Δγ^max^ increases faster than quadratic which was the main reason for our choice of the network size. Smaller networks, however, are less suitable to store small activity patterns, see section 3.2.

Apart from that, we consider several trials for each number of patterns *M* stored in the network, where each trial uses a different set of *M* patterns. Unless otherwise stated, data points are averages over 10 trials for each *M* and error bars indicate one standard deviation from the mean. Instead of the number *M* of stored patterns in the network, we will typically use the load parameter, defined as α : = *M*/*N*.

### 3.2. Memory network

**Figure 2:**
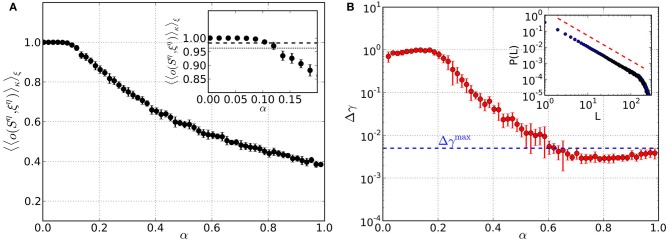
**Performance of pure memory networks in terms of retrieval quality (left) and criticality (right) as a function of the load parameter α**. Synaptic weights are fixed and defined by {*W*_*ij*_}. There is neither homeostatic learning nor activity dependent synapses adaptation. **(A)** Average overlap between initially stored patterns and corresponding retrieved patterns. Averages are taken over 10 trials for each α and error bars indicate one standard deviation from the mean. **(B)** Mean squared deviation of obtained avalanche size distributions from the best-fit power law. The blue dashed line denotes the threshold Δγ^max^ = 0.005, below which avalanche size distributions can be considered critical. The inset shows an example avalanche size distribution *P*(*L*) obtained in the range of critical load parameters, along with the slope of the best-fit power law (red dashed line).

Before we study networks that include mechanisms to bring about criticality, we first test pure memory networks. We generate a set of *M* random binary patterns, calculate the matrix {*W*_*ij*_} according to Equation 6 and set the network connectivity to *J*_*ij*_ = *W*_*ij*_. The memory quality is then assessed by calculating the average overlap ${\langle {\langle o\left(S^{\eta },\;\xi^{\eta }\right)\rangle }_{\kappa }\rangle }_{\xi }$ between the retrieved patterns *S*^η^ and the original patterns ξ^η^ (see section 2.5 for details). From here on, we always take the average 〈.〉_κ_ over *n*_*p*_ = 1000 randomly generated perturbations κ^η^ of each of the *M* patterns ξ^η^. The average overlap is close to 1 up to load parameters α ≈ 0.07 (Figure 2A), indicating perfect retrieval quality of the networks. Around α ≈ 0.11 it drops below 0.982, which marks the overlap corresponding to an average deviation of one digit from the original pattern. Finally, at α ≈ 0.13 and beyond the network does not yield retrieved patterns anymore that are closer to the original patterns than the perturbations.

In Figure 2B we show results of a criticality test for pure memory networks, where we record *A*_ava_ avalanches and consider the mean squared deviation Δγ of the size distribution *P*(*L*) from the best-fit power law. Although there is no mechanism in these networks to bring about criticality in a self-organized way, we always choose the normalization *C* in Equation 6 such that 〈*W*_*ij*_〉 corresponds to the critical value of the model with fixed synaptic weights [see section 2.1 and Eurich et al. (2002)]. We find that for load parameters α ≳ 0.6 the network is critical. Below this value it is not critical because the coupling matrix {*W*_*ij*_} is too sparse, i.e., many entries are 0. Thus, pure memory networks can become critical but only in a range of load parameters where the quality of retrieval or memory is already poor. In the following sections we show that the critical regime and the memory regime can be brought into agreement by employing the optimization strategy described in section 2.6.

**Figure 3:**
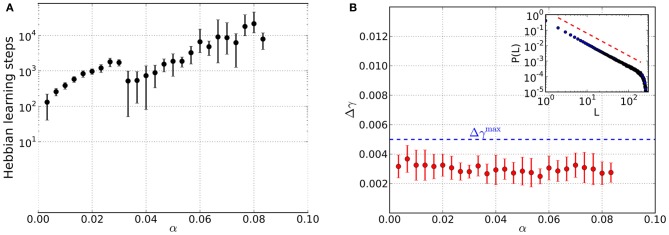
**Results from networks including Hebbian and homeostatic learning of synaptic weights, respectively, for different values of the load parameter α**. For each value of α data is taken from 10 trials and error bars mark one standard deviation from the mean. **(A)** Total number of steps in Hebbian learning needed to converge to a state that is both critical and an associative memory of the stored patterns. The discontinuity near α≈ 0.03 appears to be due to the finite size of the basins of attraction: while for low loading ratios α a basin of attraction of several bits can be achieved, now only a single bit is corrected in the course of the learning which is faster achievable than before. **(B)** Average mean squared deviation Δγ from the best-fit power law. Since all data points lie below the threshold of Δγ^max^ = 0.005 (blue dashed line), avalanche size distributions are critical over the whole range of α. An example avalanche size distribution *P*(*L*) in the converged state is illustrated in the inset (red dashed line indicates slope of the best-fit power law).

**Figure 4:**
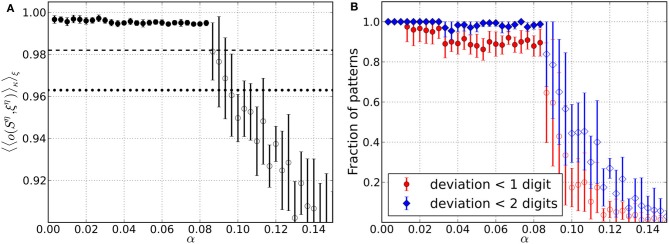
**Retrieval performance of networks including Hebbian and homeostatic learning of synaptic weights, respectively, for different load parameters α**. For each α data is taken from 10 trials and error bars mark one standard deviation from the mean. **(A)** Average overlap between initially stored patterns and corresponding retrieved patterns. Filled circles include results from converged simulations only, whereas most simulations in the range of open circles did not converge. For comparison, the overlaps corresponding to an average deviation of two digits (dotted line) and one digit (dashed line) from the original patterns are indicated. **(B)** Average fraction of patterns for which the networks yield retrieved patterns with deviation less than one and two digits, respectively. Filled markers again include converged simulations only and open markers mainly have contributions from simulations that did not converge.

**Figure 5:**
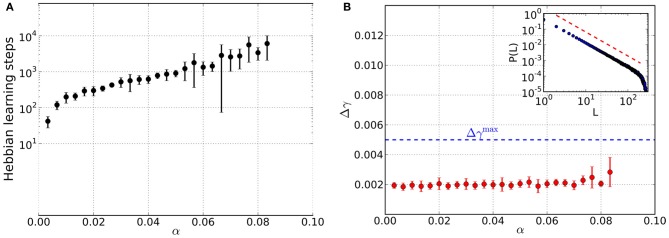
**Results from networks that are influenced by Hebbian learning and dynamical synapses, for different values of the load parameter α**. For each α data is taken from 10 trials and error bars mark one standard deviation from the mean. **(A)** Total number of steps in Hebbian learning needed to converge to a state that is both critical and an associative memory of the stored patterns. **(B)** Average mean squared deviation Δγ from the best-fit power law. Since all data points lie below the threshold of Δγ^max^ = 0.005 (blue dashed line), avalanche size distributions are critical over the whole range of α. The inset shows an example avalanche size distribution *P*(*L*) in the converged state and the red dashed line marks the slope of the best-fit power law.

The simulation time depends essentially on the load of the network, see Figures 3–5. The numerical complexity of an iteration step depends linearly on *M* as all patterns are relearned, while the other parameters on which it depends are kept fixed here.

### 3.3. Combined homeostatic and hebbian learning

We now consider simulations that include homeostatic regulation as a means to bring about criticality in a self-organized way. At the beginning, the {*J*_*ij*_} are initialized by {*W*_*ij*_} but will be modified in the course of the alternating episodes of homeostatic and Hebbian learning, respectively. The most important finding we arrive at is the existence of converged states in which the networks are critical and associative memories of the stored patterns at the same time. The total number of Hebbian learning steps needed to arrive at these states significantly increases with load parameter α (Figure 3A), spanning about two orders of magnitude. In contrast to the pure memory networks studied before, criticality is already achieved for small values of α (Figure 3B).

The retrieval quality of the networks in the converged state is again assessed by considering the average overlap ${\langle {\langle o\left(S^{\eta },\;\xi^{\eta }\right)\rangle }_{\kappa }\rangle }_{\xi }$ of the retrieved solutions *S*^η^ and the original patterns ξ^η^ (Figure 4A). For small values of α the networks are able to reconstruct the original patterns almost perfectly. However, the overlap is less than in case of the pure memory networks. Compared to the latter, the decrease in retrieval quality also occurs for smaller α and is more abrupt. The open circles mark the range of load parameters, where the majority of simulations does not converge anymore, because the required increase Δ_hebb_ in overlap during the Hebbian learning episode is not reached. Instead, the overlap saturates so that we stop Hebbian learning, add one last excursion toward the critical regime and finally finish the simulations after measuring the retrieval quality.

Since ${\langle {\langle o\left(S^{\eta },\;\xi^{\eta }\right)\rangle }_{\kappa }\rangle }_{\xi }$ only measures the overlap averaged over all the *M* patterns stored in a network, we also assess the overlap on the level of single patterns. For this reason we consider the fraction of patterns stored in a network, that can be reconstructed from perturbed states with a deviation less than a specified number of digits (Figure 4B). For the range of load parameters α where the majority of simulations converge, more than 90% of the retrieved patterns can be reconstructed with an average deviation less than one digit. (Figure 4A). Also, there are practically no patterns for which the retrieved states deviate more from the original patterns, than the perturbations themselves. Even in the range of load parameters where the average overlap strongly decreases, there is still a small fraction of patterns which is well “remembered” by the networks.

### 3.4. Synaptic depression

We now turn to the second synaptic regulatory mechanism that brings about criticality in our networks (see section 2.3). All the analysis in this part is done along the lines of the previous section, so the only essential difference here is that we substitute homeostatic learning as the mechanism that drives the network into the critical regime by dynamical synapses. At the beginning of the simulations, maximal synaptic resources $\left\{T_{ij}^{\text{max}}\right\}$ are set equal to 1.4 (*uN*)^−1^. Due to Hebbian learning however, structure in the maximal resources will develop in the course of the simulations.

Also the model networks considered here evolve toward converged states that are critical and an associative memory at the same time. While the total number of Hebbian learning steps needed to converge (Figure 5A) is comparable to homeostatic learning, the agreement of the avalanche size distributions with scale-free distributions is better for dynamical synapses (Figure 5B).

**Figure 6:**
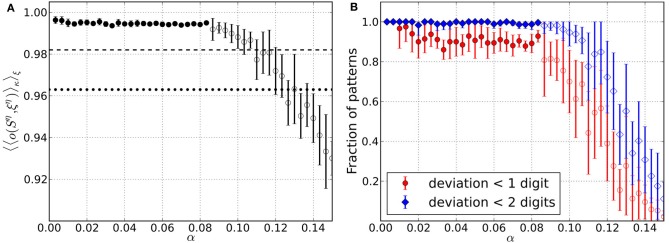
**Retrieval performance of networks including Hebbian learning and dynamical synapses for different load parameters α**. For each α data is taken from 10 trials and error bars mark one standard deviation from the mean. **(A)** Average overlap between initially stored patterns and corresponding retrieved patterns. Filled circles include results from converged simulations only, whereas most simulations in the range of open circles did not converge. For comparison, the overlaps corresponding to an average deviation of two digits (dotted line) and one digit (dashed line) from the original patterns are indicated. **(B)** Average fraction of patterns for which the networks yield retrieved patterns with deviation less than one and two digits, respectively. Filled markers again include converged simulations only and open markers mainly have contributions from simulations that did not converge.

Figure 6 addresses the retrieval quality of the converged network states for the stored patterns. The average overlap ${\langle {\langle o\left(S^{\eta },\;\xi^{\eta }\right)\rangle }_{\kappa }\rangle }_{\xi }$ is again close to optimal for small values of α and drops below the overlap of perturbed patterns 〈*o*(κ^η^, ξ^η^)〉_ξ_ at α ≈ 0.13 (Figure 6A). This is comparable to what was observed for the pure memory networks in Bibitchkov et al. (2002) so that the overlap decreases less quickly than for homeostatic regulation. This might be attributed to the fact that the structure that was learned into the maximal efficacies $\left\{T_{ij}^{\text{max}}\right\}$ during episodes of Hebbian learning is not affected during the critical phase, where only the {*T*_*ij*_} are changed. Thus, memory is safely stored within maximal synaptic efficacies.

## 4. Discussion

In this study we investigated the interplay between criticality and memory in neural networks. We showed that Hebbian learning alone destroys criticality even when the synaptic strength is properly scaled. Applying an optimization procedure that drives the synaptic couplings either toward the critical regime or toward the memory state in an alternating fashion, we finally arrive at a configuration that is both critical and retains an associative memory. In the following, we will discuss our findings and possible implications in more detail.

### 4.1. Quality of criticality

The mean squared deviation of the avalanche size distributions obtained in the converged network states from their best-fit power law was always smaller than the threshold Δγ^max^ = 0.005, providing evidence that the networks indeed operate at the finite-size equivalent of a critical point (Levina et al., 2007b). Furthermore, we did not observe oscillatory features in the avalanche size distributions. This suggests that the network dynamics circumvents the attractors (the stored patterns) that were learned into the network structure. The explanation of this observation is probably twofold. First, the majority of activity in the networks consists of small avalanches whose size is smaller than the total activity in the stored patterns, so that there is not enough overlap to be attracted toward the stored patterns. Second, even though larger avalanches have generations of firing neurons with total activity close to that of the stored patterns, the likelihood that they come close enough is very small given the many possible configurations. Indeed, we found no evidence that avalanches or their sub-generations come close to any of the stored patterns at all during our simulations. There are nevertheless traces of the pattern structures in the avalanches in the sense that a pair of neurons that is active in the same pattern is also correlated in the critical spontaneously active network. Likewise, we consider elevated correlations also between subsequent avalanches. Although these correlations are not unexpected it is interesting that they do not interfere with the criticalization of the network.

### 4.2. Quality of memory and capacity

To assess the (associative) memory quality in the converged state, we presented perturbed versions of the initially stored patterns to the networks which resulted in retrieved patterns. The latter almost never deviate from the original patterns more than the perturbed states themselves. More importantly, more than 90% of the patterns are reconstructed with on average less than one digit deviation from the original patterns. We may therefore conclude that the memory quality of the critical networks is very good. However, a pure memory network which has couplings equal to {*W*_*ij*_} and does not operate at the critical point, still performs better in terms of reconstruction from perturbed patterns. This might be attributed to the fact that the coupling matrices {*J*_*ij*_} obtained in the converged states are not symmetric anymore, as opposed to {*W*_*ij*_}. But symmetry of the coupling matrix is a major prerequisite for good retrieval quality of traditional Hopfield networks.

### 4.3. Comparing homeostatic learning and dynamical synapses

We have considered two mechanisms that can regulate a neural network toward criticality (and thus making it truly self-organized critical). Homeostatic learning regulates synaptic weights until the branching ratio approaches the critical value. Dynamical synapses on the other hand represent a biologically more justified regulatory mechanism, where the critical branching ratio is reached through the interplay of synaptic depression and recovery. In the latter model, we found that agreement between criticality and memory can only be achieved if the maximal synaptic weights are structured by Hebbian learning. Homogeneous maximal weights in contrast lead to memory loss during critical episodes because synaptic resources may recover to their maximal values which carry no information of the stored patterns anymore.

### 4.4. Memory storage by dynamical synapses

The apparent contradiction between the classical concept of memory storage by fixed synaptic efficacies which are modifiable only by persistent high-rate stimulation on the one hand, and the realization of adaptive filters based on the short-term dynamics of synaptic resources has been studied already in Tsodyks et al. (1998), Bibitchkov et al. (2002), and in more realistic models in Giudice and Mattia (2001) and Romani et al. (2006). While initially the contradiction between the two modes of operation has been studied in Bibitchkov et al. (2002), later the benefits arising from the combination were uncovered. It is interesting that the formation of memories which imply a strong structural modification in the context of attractor networks, can even be enhanced in accuracy if short-term synaptic plasticity is used in the model. The finding of critical dynamics in such networks both supports this view and expands it in the sense that a coexistence of a retrieval state and a critical exploratory state becomes possible by dynamical synapses. This suggests a solution of one of the main problems with attractor networks, namely the conditions for the escape from attractors. While this can be achieved by an additional dynamics (Horn and Usher, 1989; Treves, 2005), we have here a form of dynamics that is purely input-driven when an input is available, while it is exploratory if this is not the case. It might be interesting to consider networks with correlated patterns (see Herrmann et al., 1993) where the two effects can become intertwined.

### 4.5. Pattern-related correlations in the critical regime

One of the main points here is that the critical state serves as a ground state of the system which is assumed in the absence of specific external input. But it is, since the completely inactive state is absorbing in our model, constantly fed by spatially and temporally homogeneously distributed external noise. A specific external input has a large overlap with one of the patterns and a small overlap with all the other stored patterns. This is a necessary condition of the model, which in order to be relaxed requires a specific modification. We have dealt with such problems in our previous papers, but assume here uncorrelated patterns. In this way the overlap between any two patterns is negligible in theory. In a finite network this is not necessarily the case, but for a limited number of patterns the overlaps are smaller than the threshold for the spill-over into any of the other patterns. In a memory network below the capacity limit, the activity will therefore be confined to the pattern which is indicated by the input. The dynamics will thus not be critical. In a critical network on the other hand, we conjecture that on short time scales avalanches will be correlated to the existing patterns. Since, however, all neurons and thus all patterns receive constantly external input now, the avalanches are not confined to a pattern but will jump into other patterns on medium time scales.

### 4.6. Implications for aging and memory consolidation

Although we have not made this explicit here, the memory test can be formulated as an informational criterion (Rieke et al., 1997). The evolution of a network state toward a pattern decreases the distance and thus also the relative entropy as it is not known which bits are wrong or missing. Interestingly, the amount of information (however specified) does not improve when approaching the critical regime. This is contrary to what could be expected by considering the informational optimality of critical neurodynamics (Shew and Plenz, 2012), although the situation there is not comparable to the present attractor dynamics. The optimality concerns information capacity (Haldeman and Beggs, 2005) in the sense that at criticality the entropy of the state space is maximal (Ramo et al., 2007) with respect to a control parameter, i.e., the state of the network returns only rarely to states visited earlier. Obviously, this property is detrimental to the attractor dynamics of a Hopfield model, which pins the state near or at a certain memory state. In large networks, the number of states far away from any memory state is very large such that for moderate load a critical dynamics is possible. For low memory load the dynamics stays preferentially inside the patterns (Dasgupta and Herrmann, 2011) but is similarly expected to have an entropy maximum near criticality in the absence of a bias toward one of the patterns.

The network considered here can be characterized by the interplay between the attractor dynamics in memory retrieval and critical dynamics that provides optimal exploration of the state space. In a system like the human brain, where the number of memories increases for a large part of the personal history, it seems that there must be eventual a consequence for the flexibility and the ability to explore new patterns (Schrobsdorff et al., 2009). Considering, however, that the breakdown of memory at the critical capacity is not likely to be realistic, the conclusion that cognitive effects of aging can be explained by the effect of memory-dependent structure in the network on critical dynamics in the network does not immediately follow from the present model.

Although we could show here that memory storage and criticality are not irreconcilable, our results support a view that has been adopted by an increasing number of researchers in the last decade, namely that memory traces are not necessarily point attractors but more general dynamics objects (Herrmann et al., 1995; Natschlaeger et al., 2002; Rabinovich et al., 2008). In these approaches stability of the memories leads to a reduction of the capacity, but there may be the possibility of an active stabilization of the memories not necessarily different from the regulatory mechanism involved in criticalization. The fact that the mechanism for criticalization in the network needs to be counterbalanced by a mechanism for consolidation of the memories, should thus not be surprising, but it would be interesting to identify mechanisms that achieve both goals at the same time.

## 5. Concluding remarks

We have demonstrated that criticality can be preserved in an attractor network if both a memory consolidation process and a mechanism for regulation toward criticality are present. Among the different mechanisms for the maintenance of a critical regime, we found that the dynamics of synaptic resources is both biologically realistic and effective for criticalization, while their effect on memory capacity is moderate. Other mechanisms are possible, but are less easily biologically justifiable and have eventually a disastrous effect on the memory content unless actively counteracted. It is necessary to consider the present results in more realistic network models and under more general learning paradigms in order to understand better their significance for the natural and pathological development of biological neural systems.

### Conflict of interest statement

The authors declare that the research was conducted in the absence of any commercial or financial relationships that could be construed as a potential conflict of interest.
